# Brain age gap in multiple sclerosis: associated with disability but independent of serum biomarkers

**DOI:** 10.1177/17562864261458516

**Published:** 2026-06-23

**Authors:** Marc Pawlitzki, Patricia Kirschner, Lars Masanneck, Ramona Hagler, Elias Meier, Marius Vach, Hernan Inojosa, Tjalf Ziemssen, Vivien Lorena Ivan, Shammi More, Kaustubh Patil, Parnian Firouzi-Memarpuri, Julian Caspers, Jonathan Repple, Udo Dannlowski, Michael Khalil, Sven G. Meuth, Christian Rubbert

**Affiliations:** Department of Neurology, Heinrich-Heine University Duesseldorf, Moorenstraße 5, Duesseldorf 40225, Germany; Department of Neurology, Medical Faculty University Hospital Düsseldorf, Düsseldorf, Germany; Department of Neurology, Medical Faculty University Hospital Düsseldorf, Düsseldorf, Germany; Department of Neurology, Medical Faculty University Hospital Düsseldorf, Düsseldorf, Germany; Hasso Plattner Institute, University of Potsdam, Potsdam, Germany; Department of Neurology, Medical Faculty University Hospital Düsseldorf, Düsseldorf, Germany; Department of Neurology, Medical Faculty University Hospital Düsseldorf, Düsseldorf, Germany; Department of Diagnostic and Interventional Radiology, Medical Faculty and University Hospital Düsseldorf, Heinrich-Heine-University Düsseldorf, Düsseldorf, Germany; Department of Neurology, Center of Clinical Neuroscience, University Hospital Carl Gustav Carus, TUD Dresden University of Technology, Dresden, Germany; Department of Neurology, Center of Clinical Neuroscience, University Hospital Carl Gustav Carus, TUD Dresden University of Technology, Dresden, Germany; Department of Diagnostic and Interventional Radiology, Medical Faculty and University Hospital Düsseldorf, Heinrich-Heine-University Düsseldorf, Düsseldorf, Germany; Institute of Neuroscience and Medicine, Brain & Behaviour (INM-7), Research Centre Jülich, Jülich, Germany; Institute of Systems Neuroscience, Medical Faculty, Heinrich Heine University Düsseldorf, Düsseldorf, Germany; Institute of Neuroscience and Medicine, Brain & Behaviour (INM-7), Research Centre Jülich, Jülich, Germany; Institute of Systems Neuroscience, Medical Faculty, Heinrich Heine University Düsseldorf, Düsseldorf, Germany; Department of Dermatology, University Hospital Düsseldorf, Medical Faculty, Heinrich-Heine-University, Düsseldorf, Germany; Department of Diagnostic and Interventional Radiology, Medical Faculty and University Hospital Düsseldorf, Heinrich-Heine-University Düsseldorf, Düsseldorf, Germany; Department for Psychiatry, Psychosomatic Medicine and Psychotherapy, University Hospital, Goethe University Frankfurt, Germany; Goethe University Frankfurt, Cooperative Brain Imaging Center - CoBIC, Frankfurt, Germany; Institute for Translational Psychiatry, University of Münster, Münster, Germany; Institute for Translational Psychiatry, University of Münster, Münster, Germany; Department of Psychiatry, Medical School and University Center OWL, Protestant Hospital of the Bethel Foundation, Bielefeld University, Bielefeld, Germany; Department of Neurology, Medical University of Graz, Graz, Austria; Department of Neurology, Medical Faculty University Hospital Düsseldorf, Düsseldorf, Germany; Department of Diagnostic and Interventional Radiology, Medical Faculty and University Hospital Düsseldorf, Heinrich-Heine-University Düsseldorf, Düsseldorf, Germany

**Keywords:** brain-predicted age, glial fibrillary acidic protein, multiple sclerosis, neurofilament

## Abstract

**Background:**

Multiple sclerosis (MS) is influenced by age-related brain alterations and affects cellular aging mechanisms. Machine-learning models can estimate brain-predicted age from magnetic resonance imaging (MRI) to quantify these aging-related changes.

**Objectives:**

This study examines whether the difference between predicted and chronological age (BrainAGE) relates to clinical disability and biomarkers of neuro-axonal injury in MS.

**Design:**

This study analyzed brain-predicted age from structural 3D T1-weighted MRI in 82 patients with relapsing MS enrolled in three prospective clinical trials and 30 healthy controls.

**Methods:**

BrainAGE, calculated as MRI-predicted minus chronological age, was correlated with the Expanded Disability Status Scale (EDSS), MS Functional Composite subtests, and serum neurofilament light chain and glial fibrillary acidic protein.

**Results:**

The mean chronological age of patients and healthy controls included in this study was 39.2 and 40.9 years, respectively. Patients with MS (*n* = 82) showed a higher BrainAGE (6.48 ± 6.83 years) than controls (*n* = 30; 0.69 ± 6.5 years; *p* = 0.0002). BrainAGE increased stepwise from controls to patients with EDSS < 3 and EDSS ⩾3 (*p* < 0.0001). Higher BrainAGE correlated with worse 9-Hole Peg Test (9HPT, ρ = 0.34, *p* = 0.002) and Timed 25-Foot Walk performance (T25FW, ρ = 0.23, *p* = 0.043), but not with serum neurofilament light chain (*p* = 0.68) or glial fibrillary acidic protein (*p* = 0.33). In multivariable regression models adjusting for chronological age, sex, disease duration, and disease-modifying therapy, BrainAGE remained significantly associated with EDSS, 9HPT, and T25FW performance. sNfL and sGFAP remained nonsignificant after adjustment.

**Conclusion:**

Our findings suggest that BrainAGE and serum biomarkers capture complementary aspects of MS pathology, supporting a multimodal approach to assess disease progression.

**Trial registration:**

ClinicalTrials.gov ID: SATURATE: NCT05701423, 360PMS: NCT06501950, SAFEGUIDE-MS: NCT06461481.

## Introduction

Multiple sclerosis (MS) is a chronic disease of the central nervous system marked by both focal inflammation and diffuse neurodegenerative processes. Increasing evidence suggests that age and age-associated alterations in brain structure are key drivers of MS progression.^1^ Older patients are at greater risk of accumulating disability, regardless of disease duration or relapse frequency.^2^ Importantly, disease expression in older individuals tends to shift away from inflammation-driven relapses toward more insidious progression, characterized by gradual clinical worsening and brain volume loss.^3–5^ This is paralleled in radiological findings, which show declining gray and white matter volumes, rather than increasing lesion burden.^6,7^

Strikingly, people with MS exhibit biological hallmarks of accelerated aging even at early disease stages.^8^ These include chronic low-grade inflammation—often referred to as “inflammaging”—and premature cellular senescence. Both processes are defined by persistent inflammatory signaling, accumulation of senescent cells, and disrupted immune regulation. These aging-related mechanisms are increasingly recognized as contributors to diffuse neuroaxonal damage and brain atrophy in MS and have been linked to more rapid disability progression and reduced treatment responsiveness.^9–11^

To quantify neurodegenerative changes, neuroimaging—particularly magnetic resonance imaging (MRI)—provides critical insights into the trajectory and spatial distribution of brain volume loss in both MS and healthy aging. Traditionally, studies have used longitudinal measurements of whole-brain and regional volumes derived from MRI as surrogate markers of neurodegeneration.^12,13^ While these approaches are valuable, they typically require data over extended intervals (⩾12 months), limiting their feasibility for early detection or routine clinical use.^14,15^ An emerging alternative is the brain-age paradigm, which uses machine learning models trained on neuroimaging data of healthy subjects to estimate a person’s “brain-predicted age” from a single structural 3D T1-weighted MRI. The difference between this estimate and the individual’s chronological age—termed the brain-age gap estimate (BrainAGE)—may serve as a quantitative, age-adjusted indicator of structural brain health.^16,17^

Recent work has demonstrated that BrainAGE is sensitive to MS-related brain atrophy and correlates with both baseline disability and future clinical progression.^18–20^ These findings highlight BrainAGE as a promising imaging-based biomarker for monitoring neurodegeneration and predicting long-term disease course in MS.^21^

As an additional step toward more comprehensive disease monitoring, fluid biomarkers such as neurofilament light chain (NfL) and glial fibrillary acidic protein (GFAP) have gained attention.^22,23^ NfL is widely regarded as a marker of inflammation-associated axonal injury, while GFAP has been proposed to reflect more chronic, primary degenerative processes affecting astrocytes and glial networks in the central nervous system.^24^ Together, these biomarkers offer complementary insights into MS pathophysiology.^25,26^

Considering the need for deeper phenotyping of disease mechanisms and trajectories in MS, the present study aimed to investigate the relationship between BrainAGE, clinical severity, as well as serum levels of NfL (sNfL) and GFAP (sGFAP), exploring, whether brain age estimates capture overlapping or distinct aspects of neuroaxonal and glial damage.

## Methods

### Study population

This study is a retrospective, cross-sectional analysis of baseline MRI, clinical, and serum biomarker data from patients with relapsing multiple sclerosis using data collected from three prospective clinical trials (SATURATE: NCT05701423, 360PMS: NCT06501950, SAFEGUIDE-MS: NCT06461481) and from healthy controls. The study size was determined by the number of eligible participants available during the study period. While the SATURATE and SAFEGUIDE-MS study exclusively recruited patients with relapsing MS (RMS), the ongoing 360PMS study also includes individuals with primary progressive MS according to the 2017 revised McDonald criteria. However, for the purpose of this analysis, only RMS patients were considered. Patients with an EDSS of up to 7.0 were eligible for study inclusion. All studies were conducted as single-center observational studies at the Department of Neurology, University Hospital Düsseldorf, Germany. Recruitment of patients and data collection were conducted from February 1, 2023, to October 31, 2025. Healthy control participants (HC) were recruited from an imaging study on hyaluronic acid filler application conducted by the local Department of Dermatology. All control participants were screened to exclude a history of neurological disorders based on self-report and medical history.

Disability status was evaluated using the Expanded Disability Status Scale (EDSS), a clinician-rated scale ranging from 0 (normal neurological exam) to 10 (death due to MS). Patients with MS were categorized into two groups according to their baseline EDSS (EDSS < 3 vs EDSS ⩾ 3). Functional performance was further assessed using parts from the Multiple Sclerosis Functional Composite (MSFC), which includes the Timed 25-Foot Walk (T25FW) as well as the 9-Hole Peg Test (9HPT) for both dominant and nondominant hands.

### Magnetic resonance imaging

The study participants and HC underwent imaging on a 3T Siemens Trio scanner (Siemens Healthineers, Erlangen, Germany). Structural 3D MPRAGE T1-weighted MRI was acquired with the following parameters: TR = 1.9 s, TE = 3.34 ms, TI = 900 ms, flip angle = 9°, FoV = 256 × 256 mm, sagittal plane, slices = 192, voxel size = 1 mm^3^.

### Brain age gap estimation (BrainAGE)

BrainAGE was derived using the best-performing model from More et al.^27^ which has been trained on 2953 healthy controls (18–88 years) from four large population-based studies. In essence, T1-weighted MRI scans were processed using CAT12 (v12.8) for SPM12. The pipeline included high-accuracy affine registration (accstr = 0.8), bias-field correction, and tissue segmentation, followed by spatial normalization to MNI space using optimized Geodesic Shooting (regstr = 1; 1 mm templates). Images of 1 mm isotropic resolution were modulated to preserve local gray matter volume after linear and nonlinear transformation. The resulting modulated gray matter maps were smoothed with a 4 mm full-width-half-maximum kernel, resampled to 4 mm spatial resolution, and subjected to principal component analysis for dimensionality reduction. Further details on the preprocessing and BrainAGE modeling pipeline are provided by More et al.^27^ Gaussian process regression was used to predict age, and BrainAGE was calculated by subtracting the chronological age from the predicted age.

### Laboratory measures

Blood samples were collected at baseline from all study participants using a short catheter from an antecubital vein into Monovettes (Sarstedt, Nümbrecht, Germany). Within one hour of collection, the samples were transferred to the central biobank of the Department of Neurology, University Hospital Düsseldorf. They were centrifuged at 3000 rpm for 10 min, aliquoted, and frozen at −20°C within 60 min of collection. Within 4 weeks, samples were transferred to a −80°C freezer for long-term storage until final analysis.

Quantification of sNfL and sGFAP was performed at the Central Institute for Clinical Chemistry and Laboratory Diagnostics, Medical Faculty, University Hospital Düsseldorf. Both biomarkers were analyzed using research-use-only electrochemiluminescence immunoassays (ECLIA; two-step sandwich format) on the Roche Cobas 8000 analyzer (Elecsys^®^ module), following the manufacturer’s protocols. All laboratory personnel were blinded to clinical information. *Z* scores for sNfL and sGFAP were calculated using previously published methods,^23,28^ including assay-specific normative references for the Elecsys^®^ platform.^29,30^

### Statistical analysis

Continuous variables are summarized as mean ± standard deviation (SD) or median and inter-quartile-range (IQR, Q1–Q3). Normality was assessed using the Shapiro–Wilk test, and group differences in age with the Wilcoxon rank-sum test. The correlation between BrainAGE and chronological age was assessed in patients and HC using Pearson correlation, and a linear interaction model (BrainAGE ~ chronological age × group) was fitted to test whether the age-BrainAGE relationship differed between patients and HC. Differences in BrainAGE across EDSS categories were evaluated using the Jonckheere–Terpstra test (one-sided, increasing) and Kruskal–Wallis with Dunn’s post-hoc and Bonferroni correction. In an exploratory analysis, logistic regression was used to assess the discriminative ability of BrainAGE for EDSS categories (<3 vs ⩾3), with BrainAGE as the sole predictor. The area under the receiver operating characteristic curve (AUC) was estimated with 95% confidence intervals (DeLong method). A bootstrap optimism-corrected AUC was obtained using 1000 iterations to quantify potential optimism. Univariable correlations between BrainAGE and clinical and biomarkers measures were explored using Spearman’s correlation for raw biomarker values and years since manifestation or diagnosis. Pearson’s correlation was used for BrainAGE and *Z*-scored variables. Pearson and Spearman’s correlations were used to quantify unadjusted bivariate relationships, whereas regression models were applied to assess associations while accounting for potential confounders. All tests were two-sided, except for the Jonckheere–Terpstra test, and *p* < 0.05 was considered significant. Participants with missing data for the 9HPT or the T25FW were excluded from the respective analyses. To assess associations while controlling for potential confounders, multivariable linear regression models were fitted with BrainAGE as the dependent variable. For each clinical and biomarker predictor, an age-adjusted model (including chronological age as a covariate) and a fully adjusted model (additionally including sex, years since diagnosis, and disease-modifying therapy (DMT) category) were computed. DMT was categorized as high-efficacy therapy (HET) versus other/no therapy to limit model complexity. *Z*-score analyses were performed as sensitivity analyses. Serum biomarkers were natural log-transformed for regression analyses to approximate normality of the residuals. Variance inflation factors were calculated to assess multicollinearity. As a final sensitivity analysis, BrainAGE was residualized on chronological age using the regression slope estimated in HC, and all multivariable models were repeated with residualized BrainAGE as the dependent variable without chronological age as a covariate.

### Ethical considerations

This study was approved by the Ethics Committee of the Medical Faculty Düsseldorf (No.: 2022–2030 from September 9, 2022, 2022–2035 from October 28, 2022, 2022–2033 from September 9, 2022). Written informed consent for inclusion in this research was obtained from the patients prior to inclusion into the study. The informed consent form was reviewed by the ethics committee for approval of the study, and all trial activities were in accordance with the Declaration of Helsinki.

## Results

A total of 82 patients (10.2 ± 10.5 years since diagnosis) from the three studies and 30 HC were included in the present analysis. Baseline demographic characteristics are presented in Table 1. There was no significant age difference between patients (39.2 ± 12.0 years) and HCs (40.9 ± 15.2 years; *p* = 0.78). The sex distribution did not differ significantly between patients (68% female) and healthy controls (77% female; Fisher’s exact test, *p* = 0.49).

**Table 1.** Demographic, clinical, and biomarker characteristics of patients with MS and healthy controls.

Characteristic	Control	MS
*N*	30	82
Female (*n* (%))	23 (76.7%)	56 (68.3%)
Age (mean ± SD)	40.93 ± 15.21	39.24 ± 12.0
Years since manifestation	–	11.59 ± 10.7
Years since diagnosis	–	10.2 ± 10.5
EDSS (median (IQR))	–	2 (1–3)
<3 (*n* (%))	–	60 (73.2%)
⩾3 (*n* (%))	–	22 (26.8%)
sNfL (mean ± SD)	–	6.7 ± 5.71
sNfL *Z*-scores (mean ± SD)	–	0.31 ± 1.12
sGFAP (mean ± SD)	–	81.99 ± 50.45
sGFAP *Z*-scores (mean ± SD)	–	0.47 ± 1.26
nHPT dominant (mean ± SD) [s]*	–	21.29 ± 5.52
nHPT nondominant (mean ± SD) [s]**	–	22.97 ± 6.15
T25FW (mean ± SD) [s]***	–	5.15 ± 1.89
nHPT *Z*-scores (mean ± SD)*	–	0.19 ± 0.99
T25FW *Z*-scores (mean ± SD) [s]***	–	−0.02 ± 0.69

Values are presented as mean ± SD, median (interquartile range, IQR), or number (*n*, %), as appropriate. Disease duration was calculated both since first manifestation and since formal diagnosis.

* Missing data in two patients.

** Missing data in three patients.

*** Missing data in four patients.

9HPT, nine-hole peg test; EDSS, expanded disability status scale; sGFAP, serum glial fibrillary acidic protein; sNfL, serum neurofilament light chain; SD, standard deviation; T25FW, timed 25-foot walk.

The patients were under current therapy with natalizumab (36.6%), ocrelizumab (24.4%), ofatumumab (13.4%), ublituximab (11%), cladribine (6.1%), or glatiramer acetate (1.2%), while 7.3% of patients did not receive any disease modifying therapy at the time of the study examinations. Overall, 75 patients received HET, and 7 received other or no therapy.

**Figure 1.**
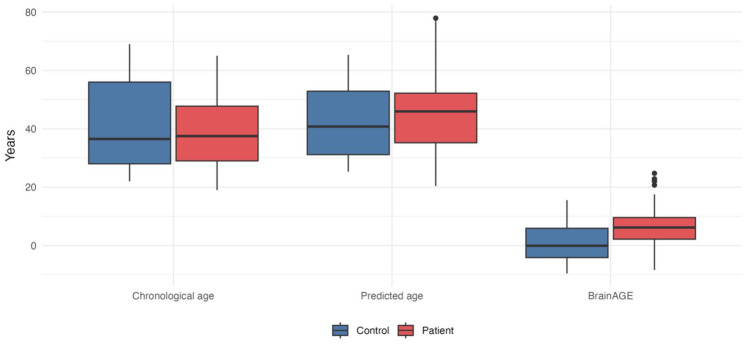
Chronological age, predicted age, and BrainAGE grouped boxplots for healthy controls (*n* = 30) and patients (*n* = 82) showing chronological age, model-predicted age, and brain age gap estimation (BrainAGE, years).

When analyzing the mean BrainAGE of the entire MS cohort (6.48 ± 6.83 years), a significantly higher BrainAGE was observed compared to the HC (0.69 ± 6.5 years), with an average difference of 5.8 years (*p* = 0.0002, Figure 1).

To assess residual age dependence of BrainAGE, we examined its relationship with chronological age separately in patients and controls. In HC, BrainAGE was negatively correlated with chronological age (*r* = −0.56, *p* = 0.001), consistent with regression-to-the-mean effects often observed in brain-age models. In patients, no significant correlation between BrainAGE and chronological age was observed (*r* = −0.03, *p* = 0.82). A linear interaction model confirmed that the age-BrainAGE relationship differed between groups (interaction *p* = 0.028; Supplemental Figure 4).

**Figure 2.**
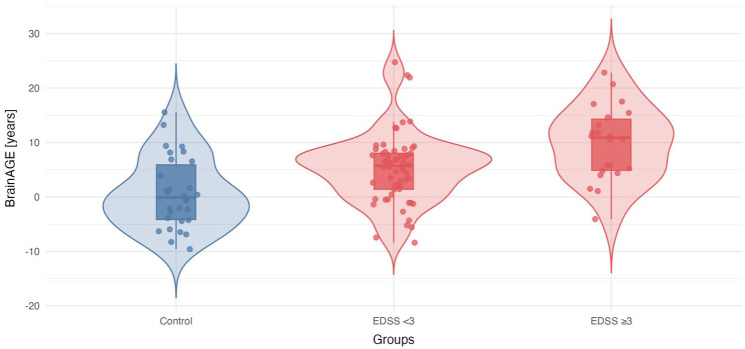
BrainAGE across EDSS-defined groups and healthy controls violin plot of brain age gap estimation (BrainAGE) by groups: healthy controls (*n* = 30), and patients with an EDSS score of < 3 (*n* = 60, 73.2%) and ⩾3 (*n* = 22, 26.8%). EDSS, expanded disability status scale.

A monotonic increase of BrainAGE from healthy controls to patients with EDSS < 3 and those with EDSS ⩾ 3 was observed (Jonckheere–Terpstra, *p* < 0.0001). The Kruskal–Wallis test confirmed overall group differences (*p* < 0.0001). Post-hoc Dunn’s tests with Bonferroni correction showed that BrainAGE was significantly higher in both patient subgroups compared with controls (Control vs EDSS < 3: *p* = 0.015; Control vs EDSS ⩾3: *p* < 0.001), and also differed between EDSS < 3 and EDSS ⩾3 (*p* = 0.040), indicating a stepwise increase across groups (Figure 2). In multivariable linear regression restricted to patients, EDSS remained significantly associated with higher BrainAGE after adjustment for chronological age (β = 1.74, 95% CI 0.75 to 2.72, *p* < 0.001), and in a model additionally adjusting for sex, years since diagnosis, and DMT group (β = 1.35, 95% CI 0.26 to 2.43, *p* = 0.015). In an exploratory logistic regression model classifying EDSS < 3 vs ⩾ 3, each additional year of BrainAGE was associated with an 11% increase in the odds of EDSS ⩾ 3 (OR = 1.11, 95% CI 1.03 to 1.21, *p* = 0.010). The area under the ROC curve was 0.69 (95% CI 0.55 to 0.83), indicating moderate discrimination, with a bootstrap-optimism-corrected AUC (1000 iterations) of 0.70.

A weak positive univariable correlation was observed between BrainAGE and years since diagnosis (ρ = 0.22, *p* = 0.047). In age-adjusted regression analysis, years since diagnosis remained significantly associated with higher BrainAGE (β = 0.25, 95% CI 0.05 to 0.45, *p* = 0.014). This finding was similar in a regression analysis adjusting for sex and DMT group (β = 0.26, 95% CI 0.06 to 0.46, *p* = 0.011). No significant correlation was found for years since manifestation in either univariable (ρ = 0.10, *p* = 0.37) or adjusted analyses (age: β = 0.16, 95% CI −0.06 to 0.37, *p* = 0.149, full: β = 0.18, 95% CI −0.04 to 0.39, *p* = 0.102).

Among the MSFC subtests, worse upper-extremity and ambulatory performance were correlated with higher BrainAGE. In univariable analyses, poorer 9HPT performance correlated with higher BrainAGE for both the dominant hand (ρ = 0.34, *p* = 0.002) and nondominant hand (ρ = 0.33, *p* = 0.003), while slower T25FW performance was also correlated with higher BrainAGE (ρ = 0.23, *p* = 0.043). In age-adjusted regression models, longer 9HPT completion times remained significantly associated with higher BrainAGE for both the dominant hand (β = 0.57, 95% CI 0.31 to 0.83, *p* < 0.001) and nondominant hand (β = 0.58, 95% CI 0.34 to 0.82, *p* < 0.001). These associations persisted in regression analyses additionally adjusting for sex, years since diagnosis, and DMT group (dominant hand: β = 0.48, 95% CI 0.20 to 0.75, *p* = 0.001; nondominant hand: β = 0.51, 95% CI 0.25 to 0.78, *p* < 0.001). Likewise, slower T25FW remained associated with higher BrainAGE after age adjustment (β = 1.37, 95% CI 0.57 to 2.16, *p* = 0.001) and in the fully adjusted model (β = 1.19, 95% CI 0.43 to 1.95, *p* = 0.003). Correlation analyses using corresponding 9HPT (*r* = −0.40, *p* < 0.001) and T25FW *Z*-scores (*r* = −0.24, *p* = 0.032) showed directionally consistent results (Supplemental Figures 1 and 2). The 9HPT Z-score was strongly associated with BrainAGE after adjustment for age (β = −3.59, 95% CI −5.08 to −2.11, *p* < 0.001) and in the fully adjusted model (β = −3.34, 95% CI −4.96 to −1.72, *p* < 0.001). The T25FW Z-score was significant in the age-adjusted model (β = −2.44, 95% CI −4.58 to −0.30, *p* = 0.026) and attenuated in the fully adjusted model (β = −1.83, 95% CI −3.99 to 0.34, *p* = 0.097).

### Serum NfL and GFAP association with BrainAGE

**Figure 3.**
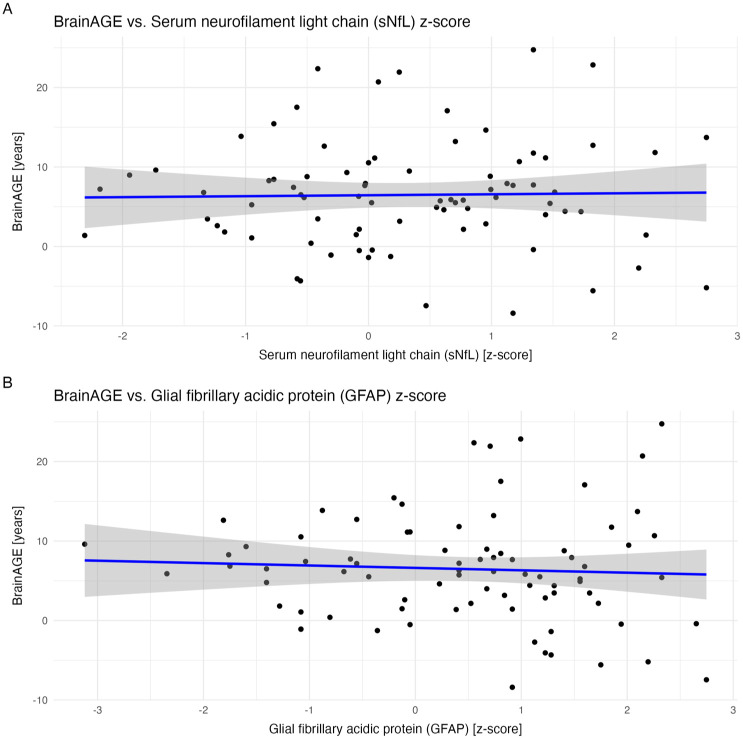
No correlation between BrainAGE and serum biomarkers. (a) Serum neurofilament light chain (sNfL) *Z* score versus brain age gap estimation (BrainAGE). (b) Glial fibrillary acidic protein (GFAP) *Z* score versus BrainAGE. Points are patients only; lines are linear fits with 95% CI.

There was neither a significant univariable correlation between BrainAGE and sNfL *Z* scores (*r* = 0.02, *p* = 0.86) nor absolute sNfL values (ρ = −0.05, *p* = 0.68). Similarly, no significant correlation was found with GFAP *Z* scores (*r* = −0.05, *p* = 0.62) or absolute GFAP values (ρ = −0.11, *p* = 0.33, Figure 3 and Supplemental Figure 3). In age-adjusted regression models, log-transformed sNfL was not associated with BrainAGE (β = −0.24, 95% CI −1.70 to 1.23, *p* = 0.75), and this finding remained unchanged in the fully adjusted model (β = 0.27, 95% CI −1.27 to 1.81, *p* = 0.73). Likewise, log-transformed GFAP showed no association in either model (age-adjusted: β = −1.05, 95% CI −3.53 to 1.43, *p* = 0.40; full: β = −0.95, 95% CI −3.38 to 1.47, *p* = 0.44).

In all multivariable models, variance inflation factors were below 2.5, indicating no relevant multicollinearity. In the sensitivity analysis using BrainAGE residualized on the healthy-control age slope, all clinical disability associations remained significant, and all biomarker null findings were confirmed, with effect sizes closely matching those of the primary analyses (Supplemental Table 1).

## Discussion

BrainAGE has recently gained attention as a radiological marker in MS. In this study, we explored its relationship with sNfL and sGFAP, two emerging biomarkers of neuroaxonal and glial injury in MS. Our results demonstrate that brain age is closely associated with clinical disability, as captured by established clinical scores, but shows no significant correlation with sNfL or sGFAP in this cohort, including in adjusted regression models. These findings may suggest that BrainAGE and serum biomarkers provide complementary perspectives on MS pathology.

Several studies have investigated BrainAGE in MS, in other neurological disorders, lifestyle risks, and healthy aging.^31,32^ It summarizes subtle, distributed structural changes into a single metric, offering advantages over regional volumetric measures. In our relapsing MS cohort, BrainAGE demonstrated a strong effect of MS on brain aging, consistent with previous studies and highlighting its sensitivity in detecting disease-related changes and the high level of brain aging compared with primary neurodegenerative disorders. In the largest and most comprehensive study to date, Cole et al.^18^ reported a correlation between BrainAGE and disease duration. In our RMS-only cohort, we observed a weak univariable correlation with years since diagnosis, which remained significant in adjusted regression analyses. Differences between studies may therefore reflect cohort composition, disease heterogeneity, and methodological variability rather than subtype-specific informativeness.

In line with previous findings, we found that BrainAGE is associated with disability, as measured by EDSS, and this relationship remained significant after adjustment for chronological age and other relevant covariates.^18^ A novel finding of our study is that BrainAGE demonstrated significant associations with functional performance measures, including 9HPT and T25FW, highlighting its potential as a sensitive MRI-based cross-sectional marker of clinical status in MS. Of note, the T25FW Z-score association attenuated to nonsignificance in the fully adjusted model (*p* = 0.097), whereas the raw T25FW time remained significant (*p* = 0.003). This likely reflects the fact that Z-scores are already age-normed and thus share less residual variance with BrainAGE once chronological age, sex, and disease duration are included as covariates. Furthermore, although age bias is a recognized feature of BrainAGE models, in our cohort, BrainAGE was negatively correlated with chronological age only in HC, whereas no such relationship was observed in patients with RMS. This suggests that the age-dependence of BrainAGE differed between groups and supports our use of age-adjusted downstream analyses. One possible explanation is that disease-related structural variation in MS attenuates the regression-to-the-mean pattern typically seen in controls, although this interpretation requires further study.

Unlike conventional volumetric measures such as brain volume loss, which have yielded inconsistent results, BrainAGE appears to provide a more robust link to clinical status.^12,14,15,27,33^ Moreover, BrainAGE may overcome technical variability between different MRI scanners and address a major limitation of current outcome measures: the reliance on longitudinal data. While MRI-based brain atrophy is an established surrogate marker, its use requires precise longitudinal assessments over at least 12 months, limiting feasibility.^12^ In contrast, we demonstrate that a single T1-weighted MRI suffices to derive BrainAGE values that capture aspects of disease-related structural changes. However, it should be noted that incremental predictive value over conventional MRI metrics was not formally assessed in the present study. BrainAGE algorithms are known to have a prediction error of up to several years in individual subjects^27^ and, therefore, results should currently be interpreted on a group level instead of a per-patient level. At the same time, the discriminative performance of BrainAGE alone for EDSS category was only modest in this cohort, also indicating that an interpretation as a standalone individual-level classifier is not suitable. Still, our results suggest that the “brain-age” framework may provide clinically meaningful information without requiring longitudinal scans, reinforcing its potential as a practical biomarker. Importantly, BrainAGE is not intended to replace established clinical measures such as EDSS or MSFC, but to complement them by providing an MRI-derived, quantitative summary of global brain structural integrity. While disability scores individualize functional impairment, they are influenced by functional reserve and compensation, relapse-related fluctuations, domain weighting (particularly for EDSS), and inter-rater variability.^34^ As a result, patients with similar EDSS/MSFC may differ substantially in their underlying neurodegenerative burden. BrainAGE addresses this gap by condensing subtle, distributed structural alterations across the brain into an intuitive unit (“years”). In cross-sectional settings, its added value lies primarily at the group level, for example, in cohort stratification and reducing heterogeneity in observational studies and trials. A lower BrainAGE (younger-appearing brain relative to chronological age) is generally consistent with less advanced structural aging at the time of assessment; however, whether this translates into improved long-term outcomes requires longitudinal validation and is not claimed based on the present cross-sectional data.

In the light of the increasing integration of the blood biomarkers sNfL and sGFAP into MS disease monitoring,^24,28,35^ we also investigated their relationship with BrainAGE. Our results show that neither sNfL nor sGFAP were associated with BrainAGE in MS, including in models adjusted for age and other relevant covariates. In a previous prospective population-based study, a correlation between BrainAGE and sNfl was found in a cohort of participants from the general population.^36^ In MS, multifaceted factors such as disease activity and treatment influence fluid biomarkers and may impact the correlation analysis. sNfL appears to more sensitively capture acute, inflammation-related neuroaxonal damage, as reflected by elevated levels at early disease stages, during relapses, or in the presence of MRI activity.^37^ By contrast, our study included only relapsing MS patients with a stable disease course for at least 6 months, drawn from three observational cohorts. Consistent with this, sNfL *Z* scores were relatively low, likely because most patients were receiving high-efficacy therapies known to substantially reduce sNfL levels.^23,28^ Regarding sGFAP, a marker thought to reflect more degenerative aspects of MS,^26^ we likewise observed no association with BrainAGE. This could suggest that sGFAP is more sensitive to microstructural damage within white matter,^22^ which may be less readily captured by BrainAGE. In addition, sGFAP appears to play a more prominent role in progressive MS,^24^ where it may provide complementary information beyond what is reflected by BrainAGE.

### Strengths and limitations

Our study has several notable strengths. Beyond previous BrainAGE analyses, we were able to demonstrate associations not only with EDSS but also with the MSFC and its subscores, thereby extending the evidence for BrainAGE as a clinically meaningful marker. This was achieved in three independent, prospectively followed cohorts of clinically stable MS patients, all examined with an identical MRI protocol on the same scanner minimizing methodological bias. Furthermore, this is, to our knowledge, the first study to systematically assess BrainAGE in relation to the blood biomarkers NfL and GFAP under standardized conditions in MS.

At the same time, some limitations must be acknowledged. Only a single BrainAGE approach on a single scanner with a harmonized protocol was evaluated in a moderately sized sample, and future studies should test different algorithms and focus on generalizability of the results in a larger cohort. Importantly, given the cross-sectional design, BrainAGE cannot distinguish between cumulative past damage, current disease activity, or individual differences in brain structure and our results must be considered descriptive. No contrast agent was applied at baseline, precluding the detection of subclinical disease activity and its potential influence on biomarkers such as sNfL. The recruitment strategy for HC may introduce a degree of selection bias, as no formal neurological examination or systematic diagnostic assessment was performed.

Different dynamics due to disease activity or treatment responses in individual patients may hinder the current analysis of a correlation between serum biomarkers and BrainAGE. The study cohort predominantly includes patients with stable RMS receiving high-efficacy therapies, which may have reduced the variability of serum biomarkers and, consequently, the ability to detect potential associations. Future studies should therefore examine BrainAGE and its relation to sNfL and sGFAP in progressive MS and untreated patients, where higher biomarker heterogeneity and cumulative neurodegeneration may provide additional insights. In addition, longitudinal analyses of clinical outcomes and serum biomarkers from these cohorts could help to further assess the relationship of sNfL and sGFAP with BrainAGE in MS.

## Conclusion

In summary, our findings reinforce the role of BrainAGE as a valuable marker of disease severity in MS, demonstrating robust associations with disability and disease duration. In contrast, no association were observed between BrainAGE and sNfL and sGFAP in this cohort. These null findings should be interpreted with caution and may reflect the specific clinical characteristics of our study population rather than fundamentally distinct biological mechanisms, that is, that BrainAGE and fluid biomarkers may capture distinct aspects of the MS pathology. These considerations highlight the importance of multimodal approaches to more comprehensively reflect disease burden and to address the concept of silent disease progression. Future data from our ongoing prospective studies could help to further explore these aspects and assess the value of BrainAGE in predicting clinical outcomes.

## Supplemental Material

sj-docx-1-tan-10.1177_17562864261458516 – Supplemental material for Brain age gap in multiple sclerosis: associated with disability but independent of serum biomarkers Supplemental material, sj-docx-1-tan-10.1177_17562864261458516 for Brain age gap in multiple sclerosis: associated with disability but independent of serum biomarkers by Marc Pawlitzki, Patricia Kirschner, Lars Masanneck, Ramona Hagler, Elias Meier, Marius Vach, Hernan Inojosa, Tjalf Ziemssen, Vivien Lorena Ivan, Shammi More, Kaustubh Patil, Parnian Firouzi-Memarpuri, Julian Caspers, Jonathan Repple, Udo Dannlowski, Michael Khalil, Sven G. Meuth and Christian Rubbert in Therapeutic Advances in Neurological Disorders

sj-docx-2-tan-10.1177_17562864261458516 – Supplemental material for Brain age gap in multiple sclerosis: associated with disability but independent of serum biomarkers Supplemental material, sj-docx-2-tan-10.1177_17562864261458516 for Brain age gap in multiple sclerosis: associated with disability but independent of serum biomarkers by Marc Pawlitzki, Patricia Kirschner, Lars Masanneck, Ramona Hagler, Elias Meier, Marius Vach, Hernan Inojosa, Tjalf Ziemssen, Vivien Lorena Ivan, Shammi More, Kaustubh Patil, Parnian Firouzi-Memarpuri, Julian Caspers, Jonathan Repple, Udo Dannlowski, Michael Khalil, Sven G. Meuth and Christian Rubbert in Therapeutic Advances in Neurological Disorders

sj-png-3-tan-10.1177_17562864261458516 – Supplemental material for Brain age gap in multiple sclerosis: associated with disability but independent of serum biomarkers Supplemental material, sj-png-3-tan-10.1177_17562864261458516 for Brain age gap in multiple sclerosis: associated with disability but independent of serum biomarkers by Marc Pawlitzki, Patricia Kirschner, Lars Masanneck, Ramona Hagler, Elias Meier, Marius Vach, Hernan Inojosa, Tjalf Ziemssen, Vivien Lorena Ivan, Shammi More, Kaustubh Patil, Parnian Firouzi-Memarpuri, Julian Caspers, Jonathan Repple, Udo Dannlowski, Michael Khalil, Sven G. Meuth and Christian Rubbert in Therapeutic Advances in Neurological Disorders

sj-png-4-tan-10.1177_17562864261458516 – Supplemental material for Brain age gap in multiple sclerosis: associated with disability but independent of serum biomarkers Supplemental material, sj-png-4-tan-10.1177_17562864261458516 for Brain age gap in multiple sclerosis: associated with disability but independent of serum biomarkers by Marc Pawlitzki, Patricia Kirschner, Lars Masanneck, Ramona Hagler, Elias Meier, Marius Vach, Hernan Inojosa, Tjalf Ziemssen, Vivien Lorena Ivan, Shammi More, Kaustubh Patil, Parnian Firouzi-Memarpuri, Julian Caspers, Jonathan Repple, Udo Dannlowski, Michael Khalil, Sven G. Meuth and Christian Rubbert in Therapeutic Advances in Neurological Disorders

sj-png-5-tan-10.1177_17562864261458516 – Supplemental material for Brain age gap in multiple sclerosis: associated with disability but independent of serum biomarkers Supplemental material, sj-png-5-tan-10.1177_17562864261458516 for Brain age gap in multiple sclerosis: associated with disability but independent of serum biomarkers by Marc Pawlitzki, Patricia Kirschner, Lars Masanneck, Ramona Hagler, Elias Meier, Marius Vach, Hernan Inojosa, Tjalf Ziemssen, Vivien Lorena Ivan, Shammi More, Kaustubh Patil, Parnian Firouzi-Memarpuri, Julian Caspers, Jonathan Repple, Udo Dannlowski, Michael Khalil, Sven G. Meuth and Christian Rubbert in Therapeutic Advances in Neurological Disorders

sj-png-6-tan-10.1177_17562864261458516 – Supplemental material for Brain age gap in multiple sclerosis: associated with disability but independent of serum biomarkers Supplemental material, sj-png-6-tan-10.1177_17562864261458516 for Brain age gap in multiple sclerosis: associated with disability but independent of serum biomarkers by Marc Pawlitzki, Patricia Kirschner, Lars Masanneck, Ramona Hagler, Elias Meier, Marius Vach, Hernan Inojosa, Tjalf Ziemssen, Vivien Lorena Ivan, Shammi More, Kaustubh Patil, Parnian Firouzi-Memarpuri, Julian Caspers, Jonathan Repple, Udo Dannlowski, Michael Khalil, Sven G. Meuth and Christian Rubbert in Therapeutic Advances in Neurological Disorders

## Declarations

**Ethics approval and consent to participate:** The Ethics Committee of Heinrich Heine University granted approval for the study (approvals no.: 2022–2030 and 2022–2033 from September 9, 2022, as well as 2022–2035 from October 28, 2022). Written informed consent for inclusion in this research was obtained from the patients prior to inclusion into the study.

**Consent for publication:** Not applicable.

**Availability of data and materials:** Further data regarding the BrainAGE analyses for this study will be shared by the corresponding author* upon reasonable request.
