# Supplementary material for: Brain age gap in multiple sclerosis: associated with disability but independent of serum biomarkers
Source: Ther Adv Neurol Disord. 2026 Jun 23;19:17562864261458516. doi: 10.1177/17562864261458516 (PMC13305517; doi:10.1177/17562864261458516)
Supplement: sj-docx-1-tan-10.1177_17562864261458516 – Supplemental material for Brain age gap in multiple sclerosis: associated with disability but independent of serum biomarkers [file sj-docx-1-tan-10.1177_17562864261458516.docx]

| **Predictor** | **β** | **95% CI** | **p** |
| --- | --- | --- | --- |
| EDSS | 1.38 | 0.31 to 2.45 | 0.012 |
| 9HPT dominant hand (s) | 0.46 | 0.19 to 0.74 | 0.001 |
| 9HPT non-dominant hand (s) | 0.50 | 0.24 to 0.76 | <0.001 |
| 9HPT Z-score | -3.15 | -4.73 to -1.56 | <0.001 |
| T25FW (s) | 1.17 | 0.42 to 1.92 | 0.003 |
| T25FW Z-score | -1.79 | -3.90 to 0.31 | 0.094 |
| sNfL (log-transformed) | 0.44 | -1.00 to 1.89 | 0.544 |
| sNfL Z-score | 0.32 | -1.01 to 1.65 | 0.635 |
| sGFAP (log-transformed) | -0.55 | -2.85 to 1.76 | 0.637 |
| sGFAP Z-score | -0.41 | -1.60 to 0.78 | 0.494 |

**Supplementary Table 1: Sensitivity analysis using residualized BrainAGE.** Multivariable linear regression models with BrainAGE residualized on the healthy-control chronological age slope as the dependent variable. Models adjusted for sex, disease duration (years since diagnosis), and disease-modifying-therapy group (high-efficency-therapy vs. other/no therapy). Chronological age was not included as a covariate, as its effect was removed by residualization. CI = confidence interval; EDSS = Expanded Disability Status Scale; 9HPT = Nine-Hole Peg Test; T25FW = Timed 25-Foot Walk; sNfL = serum neurofilament light chain; sGFAP = serum glial fibrillary acidic protein. Serum biomarkers were natural log-transformed or expressed as Z-scores.
