# Supplementary figures and images for: Brain age gap in multiple sclerosis: associated with disability but independent of serum biomarkers
Source: Ther Adv Neurol Disord. 2026 Jun 23;19:17562864261458516. doi: 10.1177/17562864261458516 (PMC13305517; doi:10.1177/17562864261458516)

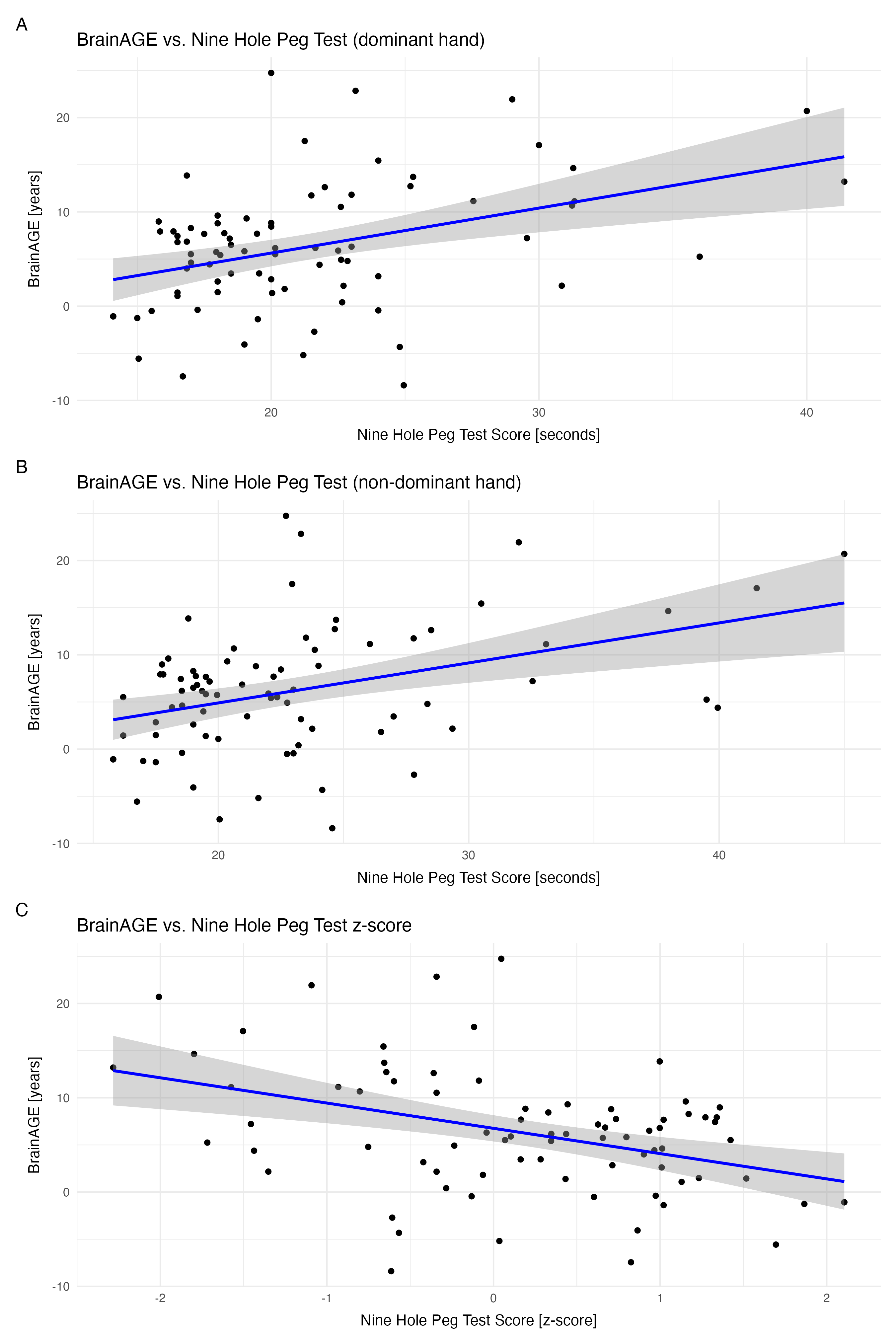

Supplement: sj-png-3-tan-10.1177_17562864261458516 – Supplemental material for Brain age gap in multiple sclerosis: associated with disability but independent of serum biomarkers [file sj-png-3-tan-10.1177_17562864261458516.png]

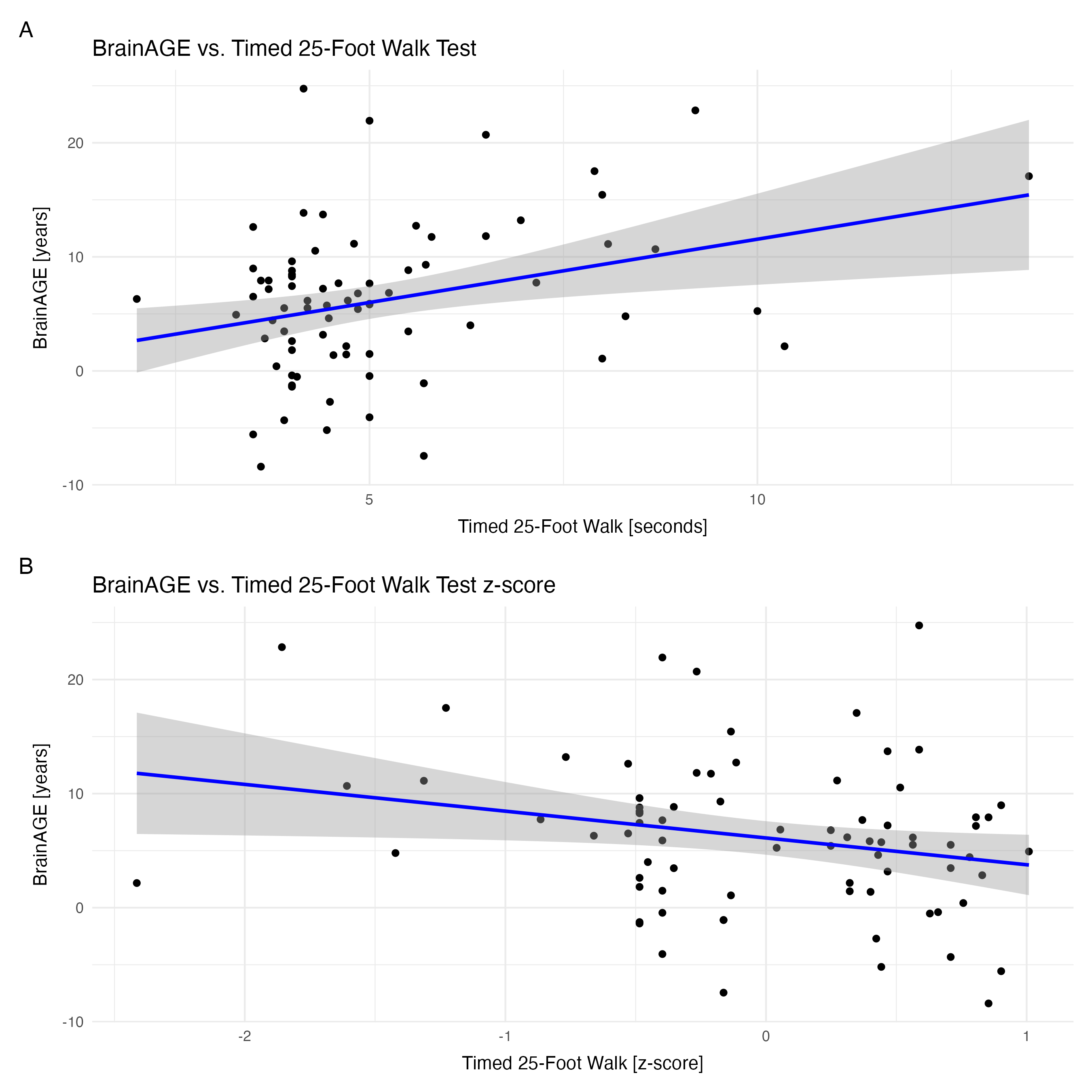

Supplement: sj-png-4-tan-10.1177_17562864261458516 – Supplemental material for Brain age gap in multiple sclerosis: associated with disability but independent of serum biomarkers [file sj-png-4-tan-10.1177_17562864261458516.png]

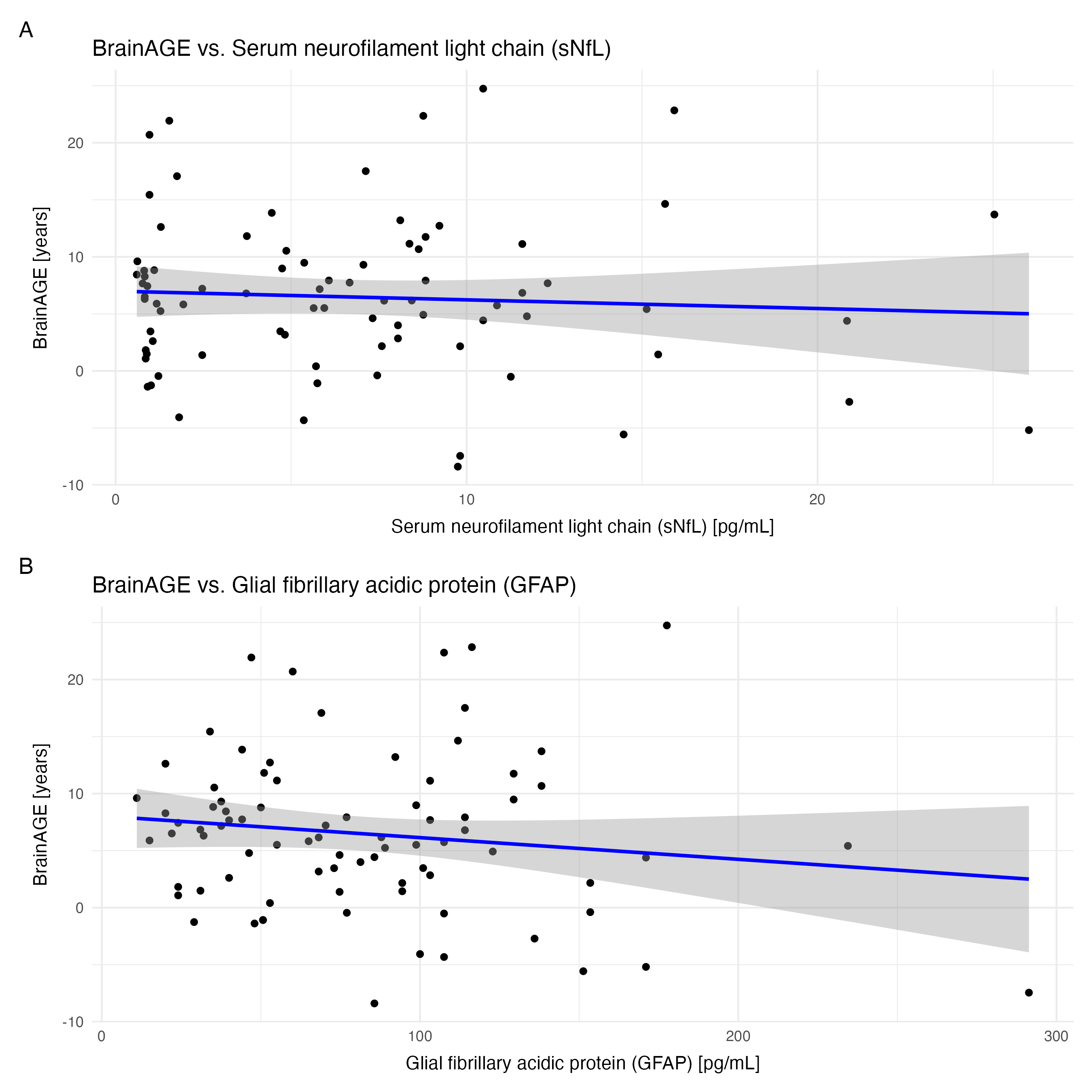

Supplement: sj-png-5-tan-10.1177_17562864261458516 – Supplemental material for Brain age gap in multiple sclerosis: associated with disability but independent of serum biomarkers [file sj-png-5-tan-10.1177_17562864261458516.png]

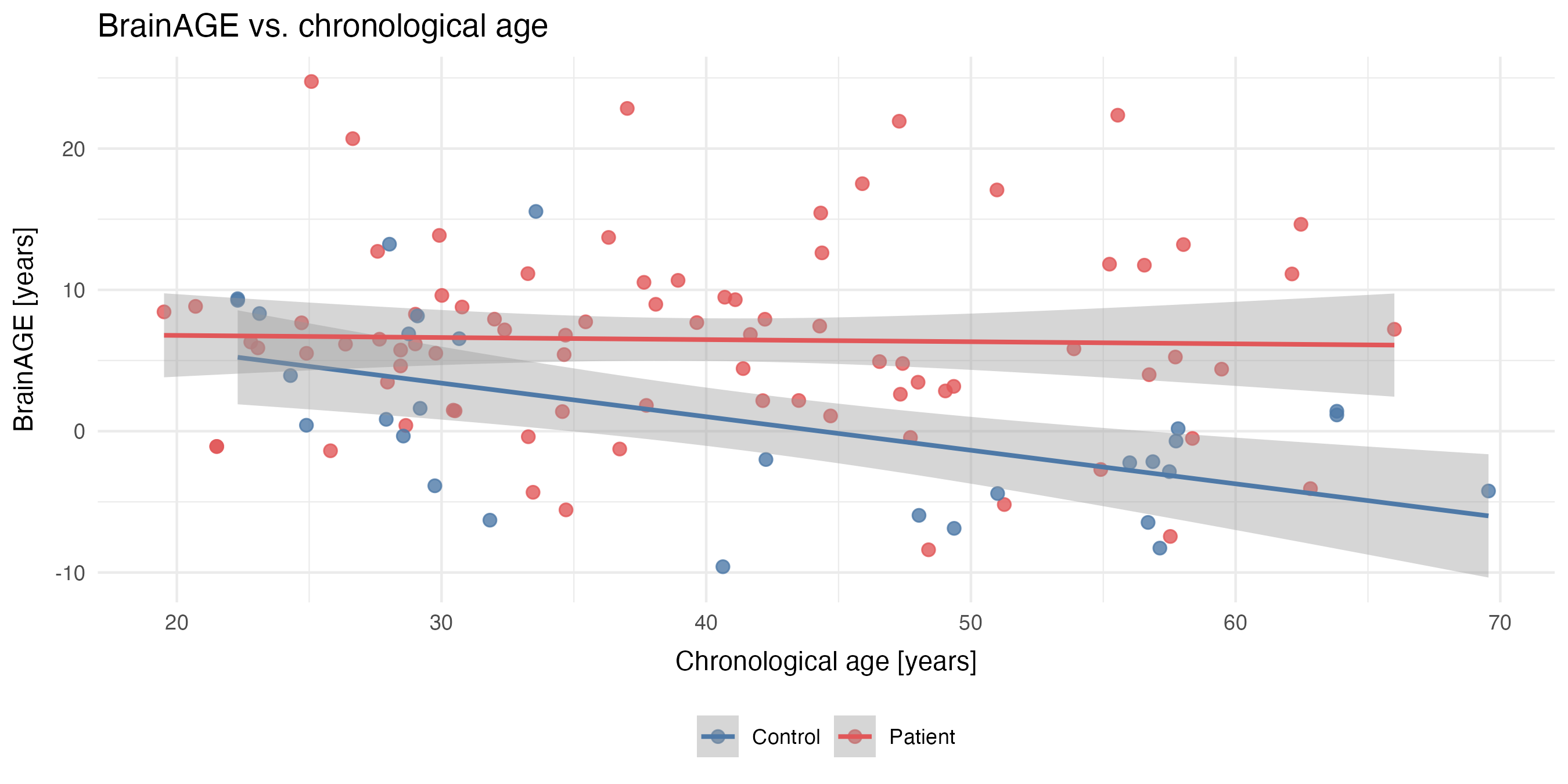

Supplement: sj-png-6-tan-10.1177_17562864261458516 – Supplemental material for Brain age gap in multiple sclerosis: associated with disability but independent of serum biomarkers [file sj-png-6-tan-10.1177_17562864261458516.png]
